# GIS integrated Mental Health Service Delivery Mapping of Korail Slum

**DOI:** 10.1192/j.eurpsy.2025.1627

**Published:** 2025-08-26

**Authors:** D. T. Rashid Soron, K. S. Azmery, T. Consortium

**Affiliations:** 1 Telepsychiatry Research and Innovation Network, Dhaka, Bangladesh

## Abstract

**Introduction:**

About one million people in Bangladesh are living in different slums in Dhaka and Korail is the largest slum of the city with more than 250000 residents. These slums are deprived from most of the basic services and cares such as sanitation, water supply and healthcare. Moreover, the situation related to mental health care might be more disappointing. However, there is no well organized service delivery map to inform us about the existing the mental health care facilities and service provider to plan a feasible real life solution for this community.

**Objectives:**

In this research, we aimed to fill this gap by developing Geographical Information Systems (GIS) integrated service delivery mapping of the slum and get a clear idea about the existing service institutional and individual level related to mental health care and related services to design and develop effective and acceptable real life solutions in the TRANSFORM Project for the person with serious mental disorders in the slum.

**Methods:**

We followed stepwise apaprach starting from reviewing the literature and other docuements to gain detailed information about the geographical coverage, culture, institutions and services those were working in the Korail slum. After this, we visited the slum to get an idea of the key landmarks and conducted stakeholders and experts meeting about data collection framework design and drafting questionnaire. Based on the discussion, the questionnaire was pretested and finalized. Then this drafted questionnaire was transferred into KoBo Collect for data collection. We tested the feasibility of KOBO tools before final data collection. We recruited the data collector from the local community and trained them to collected service institutions and service providers information with GPS locations captured with 5m accuracy level in 2022. Data was organized and analyzed using Microsoft excel and a KML file was created.

**Results:**

We found 66 Pharmacies, 50 Schools, 24 Traditional Healers, 20 Religious Institutions (mosjid mondir), 19 Madrasas, 66 medicine store, 13 health care provider chambers, 5 NGOs’ office and 2 private clinics inside the slum. Among 24 traditional healers, 20 were male and all the four female traditional healers had no formal education. The 19 faith based healers (9 madrasa teachers and 10 Imam & muajjin) provide healing practice (Jhar-fook/ panipara/tabiz/ Tadbir etc.). The community health workers of different NGOs work in the slum however, many organizations did not had their official set up in the slum. Moreover, during the service delivery mapping, we found no established mental health service in korail.

**Conclusions:**

GIS based service delivery mapping helps us in a deeper understanding of the community and to design and implement real-life mental health solutions.

**Disclosure of Interest:**

None Declared

